# Geometric Accuracy and Mechanical Property Enhancement of Fe-Based Alloy Layers in Wide-Beam Laser Direct Energy Deposition

**DOI:** 10.3390/ma18184350

**Published:** 2025-09-17

**Authors:** Bin Hu, Junhua Wang, Junfei Xu, Qingyang Wang, Li Zhang

**Affiliations:** 1 College of Physics and Electronic Engineering, Xinyang Normal University, Xinyang 464000, Chinazhangli1272@163.com (L.Z.); 2 School of Mechatronics Engineering, Henan University of Science and Technology, Luoyang 471003, China; wangjh@haust.edu.cn; 3 State Key Laboratory of Mechanical Transmission for Advanced Equipment, College of Mechanical and Vehicle Engineering, Chongqing University, Chongqing 400044, China; mecha_xjf@163.com

**Keywords:** direct energy deposition, cooling rate, Fe-based alloy, z-increment, microhardness

## Abstract

Laser direct energy deposition (LDED) has been widely employed in surface modification and remanufacturing. Achieving high-precision geometries and superior mechanical properties in cladding layers remains a persistent research focus. In this study, an Fe-based alloy was deposited on an AISI 1045 substrate via a wide-beam laser cladding system. Single-track multi-layer samples were prepared with varying z-increment (Z_d_), interlayer dwell time (T_I_) and laser scanning speed (V) values. The geometry, microstructure, microhardness and wear resistance of the samples were analyzed. Experimental results showed that an estimated Z_d_ can ensure a constant standoff distance of the laser head and resulting geometric accuracy improvement. Planar grains form at the layer–substrate bonding interface and transition to columnar grains adjacently, while dendrites and equiaxed grains are distributed in the middle and top regions of the layer. The coating layer exhibits much better wear resistance and friction properties than the substrate. The cooling rate can be substantially increased by either raising V or prolonging T_I_, resulting in refined grain structures and enhanced microhardness. Real-time monitoring and controlling the mean cooling rate have been demonstrated to be effective strategies for enhancing cladding layer performance.

## 1. Introduction

Large-scale mechanical components like rolls, gears and other moving parts are typically subjected to sustained compressive forces and frictional wear. These components exhibit significant susceptibility to surface degradation, ultimately compromising the service life of equipment [1]. Therefore, surface modification technologies have been widely utilized to promote wear resistance and fatigue resistance properties. Laser direct energy deposition (LDED), recognized as an advanced coating and remanufacturing technology, has attracted great attention in recent years. Owing to high-quality energy sources, LDED demonstrates significant advantages in terms of minimal heat-affected zones (HAZs), low dilution rates, strong metallurgical bonding and exceptional material processing flexibility [2,3,4]. Near-net-shaped components with complex geometries can be easily fabricated at low cost and with high efficiency by layer-wise stacking and adaptable deposition orientation. At present, LDED has been increasingly employed in multiple industries, such as aerospace, mold manufacturing, petrochemicals and medical health [5,6,7].

LDED melts powder or wire materials on a substrate, and compared to wire-fed LDED, powder-based LDED demonstrates superior capabilities in terms of material composition control and dimensional precision [8]. Fe-based alloy powders are typically enhanced by adding alloying elements such as chromium (Cr), nickel (Ni), molybdenum (Mo), boron (B) and silicon (Si). The addition of B and Si effectively lowers an alloy’s melting point, while simultaneously forming an oxidation-resistant protective layer on the substrate, and the incorporation of Cr and Mo can notably promote coating hardness and wear resistance [9,10,11,12,13]. The primary advantage of Fe-based alloys lies in their mechanical properties that are comparable to those of nickel- and cobalt-based alloys, while offering abundant material availability and lower costs. AISI 1045 steel is a widely used material for industrial components and equipment; traditional heat treatment processes are usually employed to promote its mechanical performance. In order to obtain better surface quality, depositing Fe-based alloy powder on AISI 1045 substrates via LDED has become a predominant method [14].

To promote the geometric accuracy of LDED, lots of researchers have focused on parameter optimization and process control [15,16,17]. For multi-layer cladding, the standoff distance of the laser head governs the locations of laser and powder convergence points [18,19]. Mazzarisi et al. [20] reported that cladding height was reduced due to unbefitting standoff distances, and the maximum powder catchment efficiency was attained when powder focalized in the melt pool center. The same findings were reported by Zhu et al. [21] and Yu et al. [22] during thin-wall part fabrication: the laser became defocused and the cladding process deteriorated when the layer growth failed to match the z-increment. Therefore, maintaining a constant standoff distance is beneficial for improving geometric accuracy and material utilization efficiency, and determining a precise z-increment is particularly crucial for muti-layer LDED. Li et al. [23] utilized the initial layer height as the z-increment within an open-loop control system, achieving favorable geometric precision. The layer height exhibits a strong correlation with process parameters. Therefore, the determination of the z-increment must account for potential variations in these parameters. Regression analysis provides an effective methodology for establishing the linear correlation between process parameters and the z-increment [19]. However, this experimental determination approach exhibits limitations in terms of cost-effectiveness and generalizability [24].

Cladding layer properties can be improved via material design [25,26], while substrate preheating and active cooling constitute another widely adopted approach [27,28]. Reindl et al. [28] reported that the whole cladding layer can gain consistent hardness by adopting a substrate preheating method. By comparing air-cooled and water-cooled cladding, Zhang et al. [29] demonstrated significantly enhanced performance in water-cooled cladding layers, with refined grain structures and substantially increased hardness. In addition, interlayer peening can also achieve similar cooling rate enhancement [30]. However, such methods introduce assistive equipment that increases system complexity and uncontrollability. In contrast, process parameter modulation offers a technically simpler approach to optimize cladding layer performance.

Extending the interlayer dwell time has been widely adopted for mechanical property promotion via LDED [31,32,33]. Foster et al. [31] reported that extending the interlayer dwell time to 40 s refined the microstructure in both Inconel 625 and Ti-6Al-4V, with the latter exhibiting yield and tensile properties comparable to those of wrought plates. With an interlayer dwell time of 360 s, the elongation and hardness of LDED 316L thin-wall parts can be increased by 47% and 13%, respectively [33]. While interlayer dwell time adjustment offers operational simplicity, it concurrently decreases deposition efficiency. Some researchers have explored other approaches to change the real-time cooling rate [34,35]. Farshidianfar et al. [27,34] carried out studies on the relationship between the laser scanning speed and cooling rate. They maintained the desired high cooling rate by implementing closed-loop scanning speed control during deposition, offering a novel approach for tailoring 316L microstructures in DED processes. Nair et al. [35] employed a comparable control strategy in IN718 DED, regulating both the scanning speed and powder feeding rate to maintain cooling rates above threshold values, thereby enhancing the resultant mechanical properties.

In summary, the z-increment and cooling rate critically influence the geometric accuracy, microstructure and mechanical properties of LDED. Cooling rate regulation has attracted significant attention for fabricating high-performance materials. However, it is not very frequently reported in Fe-based alloy LDED. In this study, Fe-based alloy multi-layer cladding experiments were conducted with varying z-increments, scanning speeds and interlayer dwell times. The z-increment values were determined through simulation and analytical modeling, with their impact on standoff distance and geometric accuracy subsequently analyzed. Cooling rate enhancement was achieved through regulation of scanning speed and interlayer dwell time. The cross-sectional samples were observed to compare microstructure, hardness and friction performance.

## 2. Materials and Methods

### 2.1. Materials

In this study, Fe-based alloy powder was employed as the cladding material. The powder was prepared by the gas atomization method, with a 0.859 spherical degree, exhibiting a good flowability. The chemical composition of the powder is detailed in Table 1. The particle size distribution and powder morphology characteristics are listed in Table 2.

The substrate material was AISI 1045, which is widely used as the main body of most industrial machines. The substrate plate size was 100 × 100 × 10 mm^3^. To improve the laser absorptivity, the substrate was polished and cleaned with alcohol and acetone before the cladding experiments. The chemical composition of the substrate material is given in Table 3.

### 2.2. Experimental Setup

**Figure 1:**
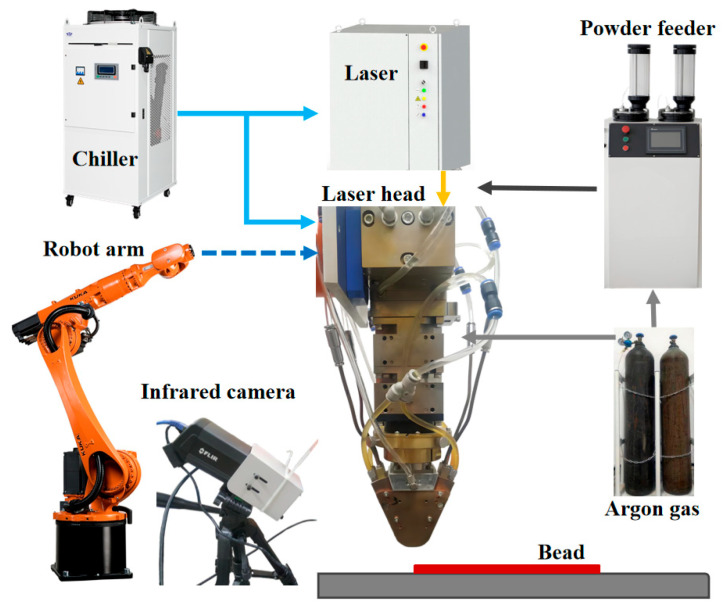
Schematic of wide-beam laser cladding system.

The experiment was carried out via a wide-beam laser cladding system (Figure 1). The wide laser beam was generated by a 3000 W fiber laser with a wavelength of 1080 nm, and the power stability was maintained at ±2%. The laser spot size was 6 × 2 mm^2^ at the focus point. The laser head was driven by a 6-axis Kuka robot arm as the end operator, and the repeat positioning accuracy was ±0.04 mm. Cooling water was pumped from a chiller to protect the laser and the laser head from overheating. The cladding material was transported by a powder feeder, whose disk rotated at 0–10 r/min. Also, 99.99% argon gas was adopted as a powder carrier gas and shielding gas. The cladding process was monitored by an infrared camera, which was installed at 60 degrees from the horizontal plane.

### 2.3. Methods

#### 2.3.1. Experimental Approach

Single-track multi-layer cladding experiments were carried out to investigate the influences of process parameters on the shape and mechanical properties of the cladding layer. The scanning speed (V), axial lifting amount (Z_d_) and interlayer dwell time (T_I_) were chosen as parameters of interest, and three cladding samples were prepared by varying the selected parameters. The laser power (P) was 2000 W, and the powder feeding rate (F) was 22.5 g/min. The powder carrier gas flow rate (Q_P_) and the shielding gas flow rate (Q_S_) remained unchanged and were 10 L/min and 12 L/min, respectively. The laser head was 18 mm away from the substrate during the first layer deposition (i.e., initial standoff distance (D_S_)). The axial lifting amount was equal to the height of a single layer, which was predicted according to our previous research [24,36]. The scanning speed (V) and the interlayer dwell time (T_I_) were changed to obtain different cooling rates. The process parameters of each sample are listed in Table 4.

Each sample was 80 mm long and had 5 layers. When one layer was finished, the laser and powder feeder were shut off immediately; then, the laser head rose up according to the preset Z_d_ and returned back to the track starting point synchronously. The next layer started after waiting for time T_I_, and the shielding gas was kept flowing during the whole process.

#### 2.3.2. Mechanical Property Analysis

The sample track was cut transversely via a wire cutting machine; a cross section was ground, polished and etched for metallographic inspection. The microstructure was analyzed by an electron microscope. The microhardness was tested along the vertical direction of the cross section, and the sampling points were from the cladding layer surface to the substrate. To evaluate the wear resistance of the cladding layer, friction-wear tests were conducted on the cladding layer and the substrate. The surfaces were ground smooth, without scratches. Silicon nitride balls were used as the friction pair, a pressure of 1000 g was applied and the grinding time was 30 min. The friction coefficient was recorded during the test, and the worn surface was scanned by a three-dimensional topography profilometer.

#### 2.3.3. Cooling Rate Monitoring

Thermocouples, pyrometers and infrared cameras are usually employed to monitor the thermal history of laser cladding. Infrared cameras can implement non-contact measurement online for the deposition process. There is a difficulty in determining the infrared emissivity due to the variation in the surroundings and the surface emissivity of the melt pool. To simplify the calculation, the infrared emissivity was generally considered as a constant value in online monitoring [34]. Thus, temperature measurement deviations were inevitably introduced, and it was necessary to validate the reliability of the temperature measurement. Infrared images can measure the melt pool size with considerable precision based on temperature gradients, which has been validated via combined experimental and simulation approaches in our prior work [24]. Therefore, despite the presence of emissivity errors, infrared images can accurately measure the cooling rate on melt pool surfaces. In this work, thermal video streams were recorded by an infrared camera, and the cooling rate was calculated according to the method proposed by Farshidianfar et al. [34,37]. The images were 800 × 600 pixels, where each pixel corresponded to the real-time radiation temperature. The picture was converted to an 8-bit grayscale image, and the temperature of the pixels was determined based on the gray value between 0 and 255.

**Figure 2:**
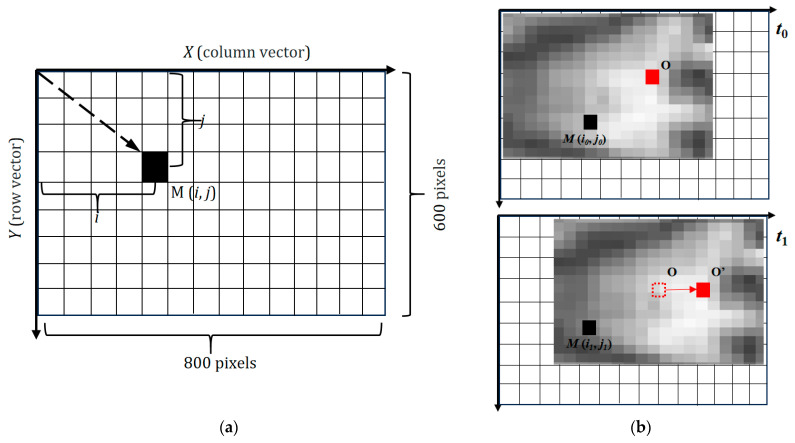
Method of cooling rate online monitoring via infrared images. (**a**) Pixel array distribution in an infrared image of A615. (**b**) Cooling rate measurement of a fixed location on the cladding track.

Figure 2a shows the two-dimensional distribution of the pixel array, and X and Y are the numbers of rows and columns, corresponding to 800 × 600 pixels. The location of each pixel can be expressed as M (*i*, *j*), where *i* is the column vector and *j* is the row vector. The main region of interest (ROI) is the melt pool, whose occupied pixels should be increased as much as possible in the infrared image. A practical approach is to reduce the view field of the camera; in the experiment, the camera was installed approximately 30 cm away from the deposition position.

The pixel with the maximum gray value has the highest temperature, which can be considered as the center of the melt pool. The method of measuring the cooling rate online via an infrared image is illustrated in Figure 2b. Two frames of images were captured at different times (t_0_ and t_1_). The points O and M are the melt pool center and a fixed location on the cladding track, respectively. At time t_0_, the first image was recorded by the infrared camera, where O is the melt pool center, M represents another point on the melt pool whose location is marked as M (i_0_, j_0_), and the temperature of M is T_0_ at this moment. At t_1_ the next image was captured, and the melt pool had moved a certain distance relative to the previous image. The melt pool center (O) was replaced by O’ in the new image. The position vector of M was unchanged, and the location was marked as M (i_1_, j_1_). The temperature of M dropped to T_1_ due to cooling down. The cooling rate of any location (M) on the melt pool can be expressed as follows [34]:

(1) $$C_{M}(t)=\left|\frac{T_{1}-T_{0}}{t_{1}-t_{0}}\right|,$$

It is worth noting that the infrared image only offers the radiation temperature of the melt pool surface, which deviates from the actual temperature. However, the measurement error has no influence on the calculation of the cooling rate, according to Equation (1). Furthermore, the calculated cooling rate is only for the locations on the melt pool surface; the inner cooling rate of the melt pool is unobtainable.

## 3. Results and Discussion

### 3.1. Analysis of Morphological Characteristics

**Figure 3:**
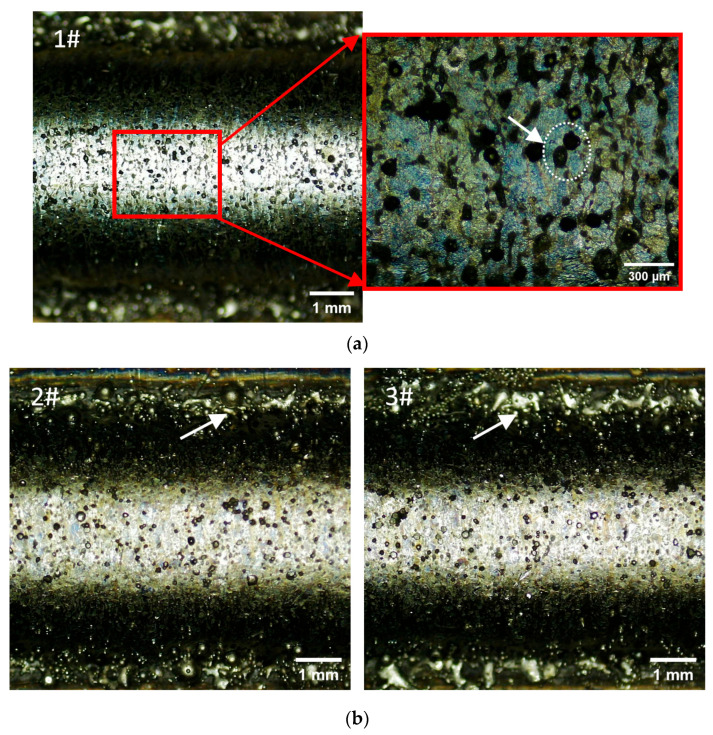
Macroscopical observation of the samples. (**a**) Powder ablation and oxidation (marked by dotted circle and white arrow) on the surface of Sample 1. (**b**) Agglomerate powders (marked by white arrow) on the track edges of Samples 2 and 3.

The macroscopic features of the samples are displayed in Figure 3. No obvious voids or defects were observed on the surface of any of the samples. A few ablative powders adhered to the surface of the cladding layer, and many agglomerate powders could be found on both sides of the cladding track, where poor metallurgical bonding with the substrate was exhibited. The cladding layers on the top became narrower than those on the bottom, as the laser spot was wider than the cladding track; therefore, the powder remaining near the track was melted inadequately by the edge of the laser beam at a low intensity. As can be seen in Figure 3a, some black particles were distributed on the bead surface; this was due to the ablation and oxidation of the powder. In Figure 3b, the agglomerate powders on the track edge of Sample 3 are more abundant than those on Sample 2. This is because the residual powder is more difficult to melt sufficiently in the case of the long cooling time for the substrate (50 s).

### 3.2. Cross-Sectional Analysis of the Cladding Layer

#### 3.2.1. Layer-Wise Thickness and Axial Lifting Amount

**Figure 4:**
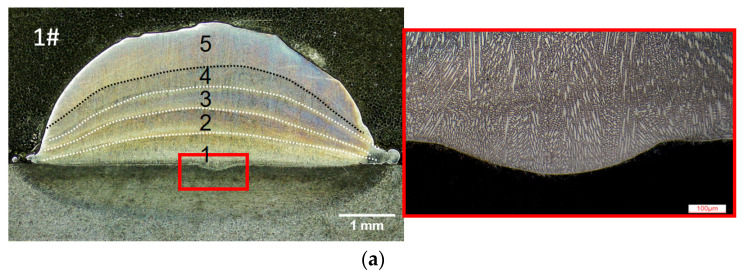

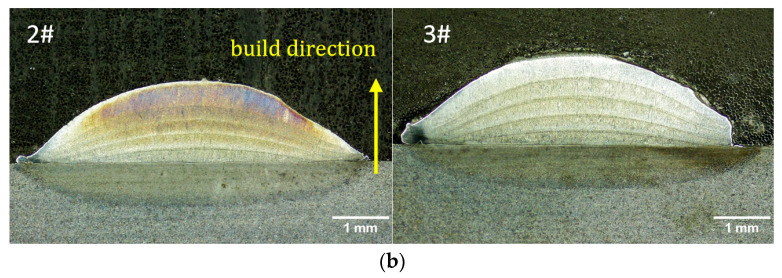
Cross-sectional morphology of the single-track multi-layer cladding. (**a**) Fusion lines and metallurgical bonding of Sample 1. (**b**) Cross sections of Samples 2 and 3.

The cladding layer was sectioned transversely for further characterization. The resulting cross-sectional morphology of each sample is presented in Figure 4. The examination revealed no detectable defects (e.g., voids or cracks).

Due to obtaining a higher laser specific energy, Sample 1 exhibited a more pronounced dilution zone compared to the other samples, indicating good metallurgical bonding with the substrate. Distinct fusion lines could be found across all the samples. The fusion line was generated due to reheating and remelting of the pre-built layer, corresponding to a narrow band with more coarse grains than the surrounding area [38]; fusion lines have been commonly utilized as demarcation boundaries between adjacent layers in prior research [39]. Figure 4a illustrates the five layers marked by dash lines; the top layer demonstrates a significantly greater thickness compared to the underlying layers. Without experiencing remelting of the last layer, Samples 2 and 3 similarly exhibit thicker uppermost layers relative to their underlying layers. Additionally, the remelting process induces molten material flow; the uppermost layer shows greater convexity relative to the underlying layers.

**Figure 5:**
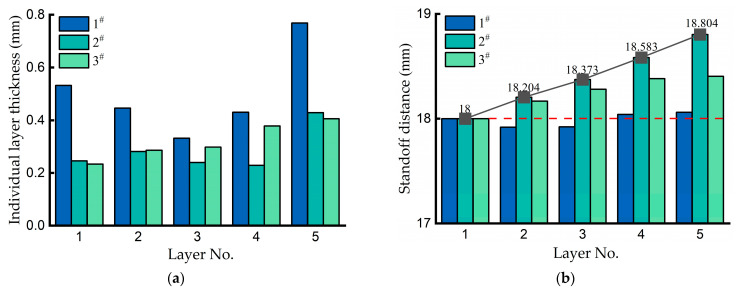
Individual layer thickness and the laser head’s standoff distance for each layer deposition. (**a**) Comparative analysis of layer thickness. (**b**) Comparative analysis of the laser head’s standoff distance (the red dashed line represents the initial standoff distance of 18 mm).

Depending on the process parameters, the samples exhibited significant variations in both total build height and individual layer thickness. Figure 5 comparatively presents the layer-wise thickness and the standoff distance of the laser head during each layer deposition. In Figure 5a, due to the lower scanning speed, each individual layer belonging to Sample 1 displays a greater thickness compared to equivalent layers in the other samples. Especially the first and fifth layers of Sample 1 demonstrate significant thickness, exceeding their counterparts in Sample 3 by 127% and 89%, respectively. Samples 2 and 3 share identical process parameters (P and V), exhibiting slight layer thickness discrepancies due to different axis lifting amounts (Z_d_).

To maintain a consistent standoff distance, the laser head needs to be lifted a specified distance before the current layer’s deposition. However, after the first layer, the thicknesses of subsequent layers are difficult to predict; thus, the first layer height has to be adopted as Z_d_ and be kept consistent in subsequent layer deposition. Z_d_ of Sample 1 was set to 0.45 mm by pre-estimation, while the Z_d_ strategy was not used in Samples 2 and 3. As illustrated in Figure 5b, all layers in Sample 1 maintained standoff distances close to 18 mm. Without the Z_d_ strategy corresponding to an increase in V, the standoff distances of Sample 2 became greater with the increase in the as-built layers, reaching 18.804 mm at the last layer. The laser beam and powder flow failed to concentrate on the focal plane, resulting in a continuous decrease in layer thickness, especially from the second to fourth layers. By adjusting Z_d_ to 0.4 mm, Sample 3 demonstrated improvements in both layer thickness and standoff distances; however, it still exhibited undesirable deviations from the expected values. This indicates that accurate axial lifting amount estimation is critical for improving forming accuracy.

#### 3.2.2. Layer-Wise Width

The initial deposition layer undergoes more rapid cooling because of the substrate’s heat dissipation effect, while it demonstrates progressively slower cooling rates due to thermal accumulation from preceding layers. Gravity-induced flow spreads molten material toward both edges of the cladding layer, resulting in wider deposition for subsequent layers compared to the initial layer. As the layers increase, a thin wall with a narrower base and a wider top is formed, presenting compromising dimensional precision.

**Figure 6:**
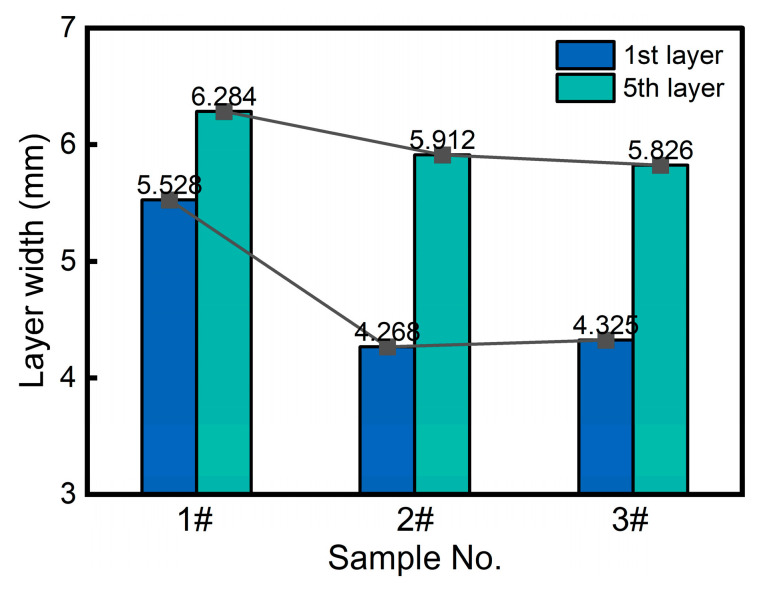
The width of the first and fifth layers of the samples.

Figure 6 presents a comparison of the results for the widths of the first and fifth layers within each sample layer. The results demonstrate a consistent pattern of width expansion in the fifth layer compared to the initial layer, with particularly significant increases observed in Samples 2 (38.5% growth rate) and 3 (34.7% growth rate). The width difference between the top and bottom layers of Sample 1 is relatively small, which might be due to the estimated axial lifting amount (i.e., the Z_d_ strategy) having improved the geometric accuracy.

### 3.3. Microstructure and Mechanical Properties

#### 3.3.1. Microstructure Analysis

It is generally recognized that the temperature gradient (G) and solidification rate (R) are pivotal parameters governing the grain morphology and dimensions in LDED [40]. The G/R ratio decides the grain morphology, and a high G/R ratio usually generates a planar grain; as R increases, the grains gradually transform to cellular, columnar and equiaxed grains. Moreover, G × R quantitatively defines the cooling rate which governs the grain sizes. Specifically, lower cooling rates promote coarse grain formation due to prolonged dendritic growth, whereas higher cooling rates induce grain refinement through rapid heterogeneous nucleation, contributing to enhanced mechanical properties [41].

**Figure 7:**
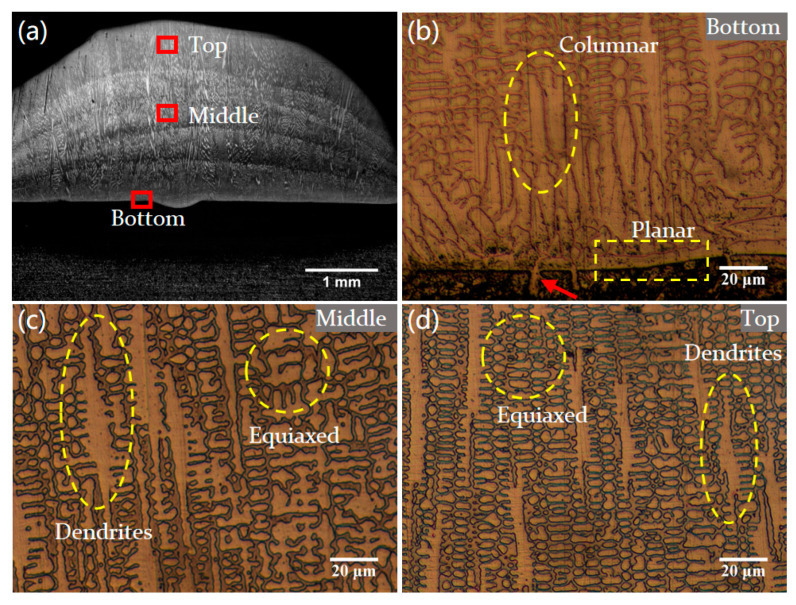
Microstructure of the cladding layer. (**a**) Different regions of Sample 1 in a cross section for microanalysis. (**b**) Bottom region of the layer. (**c**) Middle region of the layer. (**d**) Top region of the layer.

Figure 7 presents the microstructure of different regions across the cladding layer. As illustrated in Figure 7b, a metallurgical belt formed by planar grains can be found at the cladding layer bottom. The planar grains exhibit epitaxial growth along the bonding interface, with needle-like crystals penetrating the substrate (marked by red arrow), forming a characteristic intergranular eutectic structure. This indicates good metallurgical bonding between the cladding layer and the substrate [9]. Near the bonding interface, the planar grains gradually transition into columnar and dendrite structures. This is mainly attributed to the temperature gradient (G) descending from the substrate to the cladding layer; non-equilibrium interfacial solidification usually induces grain morphology transformation [42]. During multi-layer cladding, the interlayer temperature gradient exhibits a continuous decline, while heat accumulation causes a corresponding deceleration in the solidification rate, ultimately leading to a reduction in the G/R ratio. Figure 7c,d present the microstructures within the middle and top regions of the cladding layer. Both dendritic and equiaxed grains are observable, with the top region of the cladding layer exhibiting a finer microstructure.

#### 3.3.2. Microhardness Analysis

**Figure 8:**
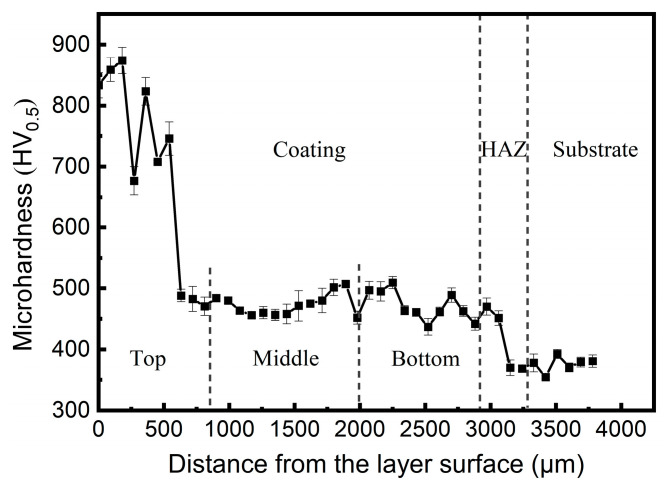
Cross-sectional mircohardness curve of Sample 1 (averages ± σ). The test direction is perpendicular to the bonding interface toward the substrate.

Microhardness tests were performed on the cross section of Sample 1. As shown in Figure 8, the microhardness varies significantly across different regions. Notably, the maximum value of approximately 900 HV_0.5_ can be observed at the layer surface, while the substrate displays the minimum hardness value of merely 300 HV_0.5_. The surface hardness of the cladding layer exhibits a remarkable enhancement, approximately three times higher than that of the substrate. The high microhardness is attributed to the finer grains formed under the low G/R ratio [43]. This substantial improvement conclusively demonstrates the effectiveness of the Fe-based cladding layer in significantly strengthening the mechanical properties of the substrate.

The middle and bottom regions of the cladding layer exhibit a relatively constant microhardness distribution, with slight fluctuations at the interlayer. This is probably attributable to the grain refinement of the fusion lines [39], exhibiting a distinct hardness enhancement. The coating layer, HAZ and the substrate can be clearly distinguished according to the microhardness curve. The high hardness of the layer surface greatly enhances its wear resistance, whereas the interior layer shows a uniform dendritic microstructure with a homogenous hardness distribution. The results confirm that the selected process parameters are well-optimized, contributing to superior cladding layer performance.

#### 3.3.3. Wear and Friction Analysis

**Figure 9:**
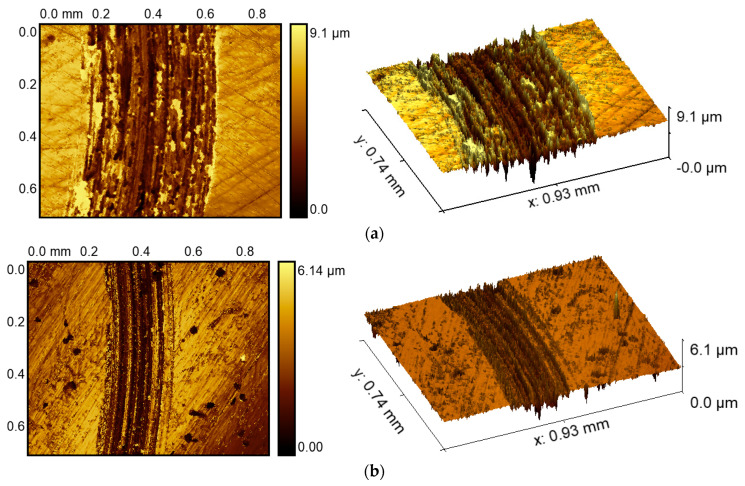
Wear morphology of the substrate and cladding layer. (**a**) Wear tracks and 3D morphology of the substrate. (**b**) Wear tracks and 3D morphology of the cladding layer.

Comparative results for wear morphology reveal differences between the substrate and the cladding layer in Figure 9. Due to the lower hardness, the substrate demonstrates significantly increased wear width and depth, exhibiting irregular abrasive tracks along the friction direction with pronounced surface deformation. The 3D topography exhibits a non-uniform wear depth distribution on the substrate surface, with the maximum wear depth reaching 9.1 μm, exceeding about 50% of the coating’s wear depth.

The friction between abrasive materials and metal surfaces typically manifests as dry friction, with wear primarily occurring through material transfer and accompanied by the formation of wear debris. Furthermore, the hard abrasive particles mix into the metal surface under the normal force and consequently move along the tangential direction of the parallel force. This caused shear deformation and winkled morphology on the substrate surface. Plastic deformation displaces material within the plough laterally, resulting in accumulations forming characteristic ridges at groove boundaries. Under repeated friction, fatigue fracture and adhesion to the metal surface occurred. This indicates that substrate wear predominantly comprises pronounced adhesive wear and abrasive wear mechanisms. As for the coating layer, embedded wear debris and abrasive particles performed micro-cutting upon being pressed into the surface. Therefore, Figure 9b primarily presents ploughing grooves.

**Figure 10:**
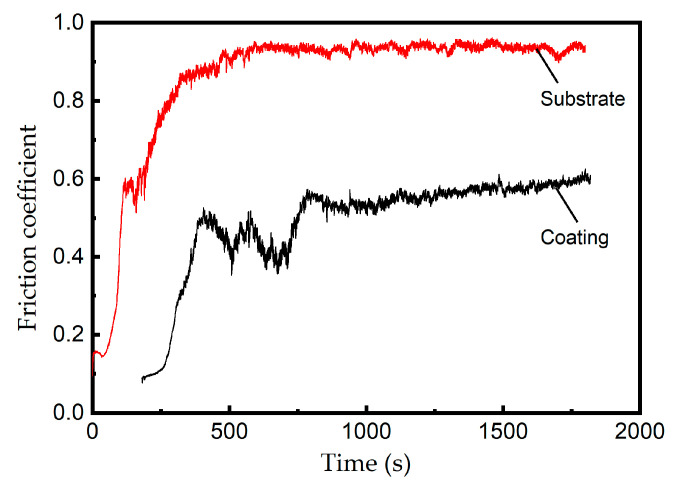
Friction coefficient–time curves of the substrate and cladding layer (the variance of the three repeated tests is 0.002).

Comparative results for the friction coefficient between the substrate and cladding layer are presented in Figure 10. In the initial friction stage (0–400 s), the substrate friction coefficient rapidly increases from 0.2 to 0.87, while the coating friction coefficient gradually increases from near 0 to 0.5. During the intermediate phase (400–1500 s), the substrate maintains a stable high-friction state at approximately 0.92 with minimal fluctuations, while the coating displays a pronounced fluctuation amplitude as its coefficient slowly ascends to 0.6. In the final stage (>1500 s), both materials reach equilibrium states—the substrate stabilizes at ~0.9, the coating at ~0.56.

The cladding layer shows a markedly lower friction coefficient than the substrate. Within a short duration, the substrate surface was sheared with grooves by the hard materials of the friction pair. The plastically deformed material adhered to the surface and was subsequently worn off, resulting in an initial decrease in the friction coefficient within a few minutes. As adhesion progressed, a stable friction regime was established, resulting in the cessation of friction coefficient fluctuations. Abrasive particles broke apart and embedded in the hard coating surfaces, resulting in a rapid increase in the friction coefficient. Then, as these abrasive particles penetrated deeper and were gradually worn smooth, the friction coefficient decreased. With repeated cycles, the furrow count grew and stabilized, explaining the early-stage friction coefficient fluctuations of the cladding layer.

### 3.4. Cooling Rate Regulation and Microhardness Improvement

#### 3.4.1. Cooling Rate Analysis

**Figure 11:**
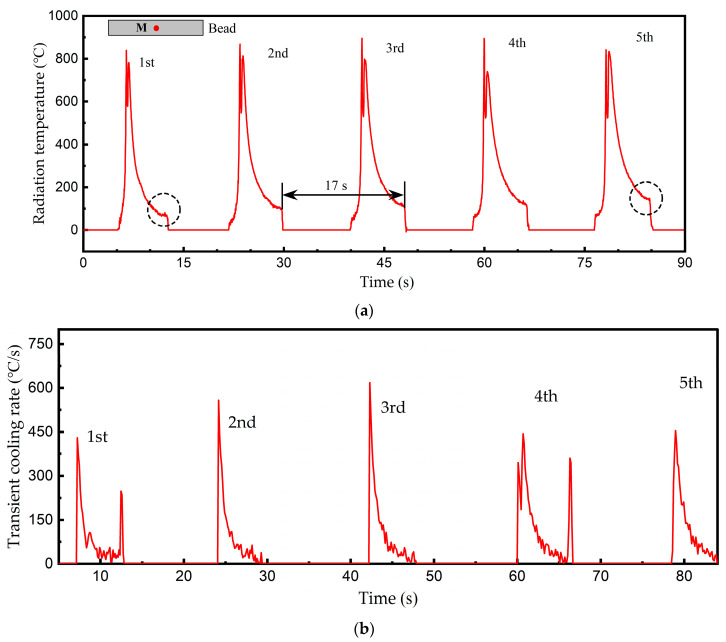
Thermal history and cooling rate of a fixed point (M) on the melt pool surface in multi-layer cladding processing (Sample 1). (**a**) Radiation temperature profiles of M on each cladding layer. (**b**) Calculated transient cooling rate of M according to the radiation temperature.

Figure 11a presents the infrared temperature curves of the Fe-based alloy multi-layer cladding progress. The bead was 50 mm long, and a location (M) was chosen in the bead middle. The infrared images of each layer were recorded, and the radiation temperature of M was extracted. As the melt pool passed, M experienced a rapid temperature rise and consequent cooling down, with an interlayer cooling interval of 17 s. Due to heat accumulation, the first layer had the lowest temperature at the end of cooling. The temperature of the subsequent layers rose gradually; the last layer was 67 °C higher than the initial layer (see dashed circles in the Figure 11b). As can be seen in Figure 11b, the transient cooling rate of each cladding layer was computed through analysis of the temperature descent phase, using a sampling time of 120 ms. The cooling rate initially had a significantly high value, corresponding to the steep temperature curve, and then decreased progressively. It should be noted that point M is located at the weld bead centerline (along the scanning direction). As the melt pool passed though, point M’s thermal history temperature was higher than that of the other positions far from the melt pool center, thereby inducing spatial variations in cooling rates. To systematically investigate the effects of scanning speed and interlayer dwell time on cooling rates, this study designated point M as the representative measurement location.

The transient cooling rate demonstrates a significant downward trend owing to the rapid temperature decrease, while showing high sensitivity to both measurement errors and infrared image disturbances. Furthermore, higher sampling frequencies tend to amplify the fluctuations observed in the cooling rate measurements. Therefore, the mean cooling rate of each cladding layer was computed and listed in Table 5.

**Figure 12:**
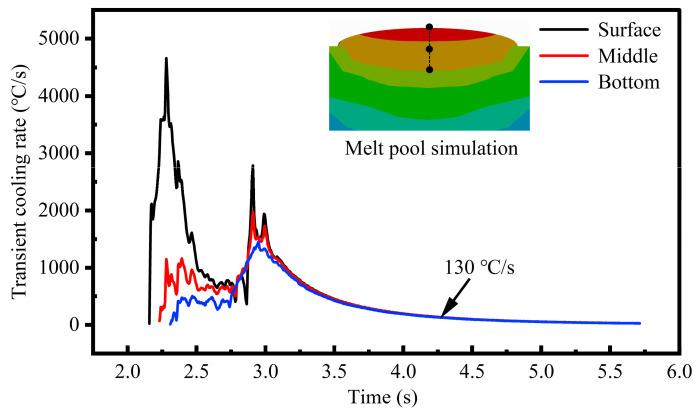
Cooling rate inside the melt pool computed through a thermal simulation model (P = 2000 W, V = 7 mm/s).

According to our developed thermal field simulation model [44], the cooling rates of various locations across the melt pool cross section were calculated for P = 2000 W and V = 7 mm/s. As shown in Figure 12, the initial high transient cooling rate and fluctuations present similar trends in the experimental results (Figure 11b). The melt pool surface demonstrates the highest initial cooling rate due to strong temperature gradients, while the interior regions present a substantial decrease in the cooling rate owing to the weakened convection. The cooling rates throughout the melt pool progressively converge to a stable value of approximately 130 °C/s, consistent with the experimental results. The analytical findings demonstrate that monitoring the melt pool surface cooling rate via online infrared imaging provides an effective indirect assessment of internal solidification dynamics.

#### 3.4.2. Cooling Rate Regulation

**Figure 13:**
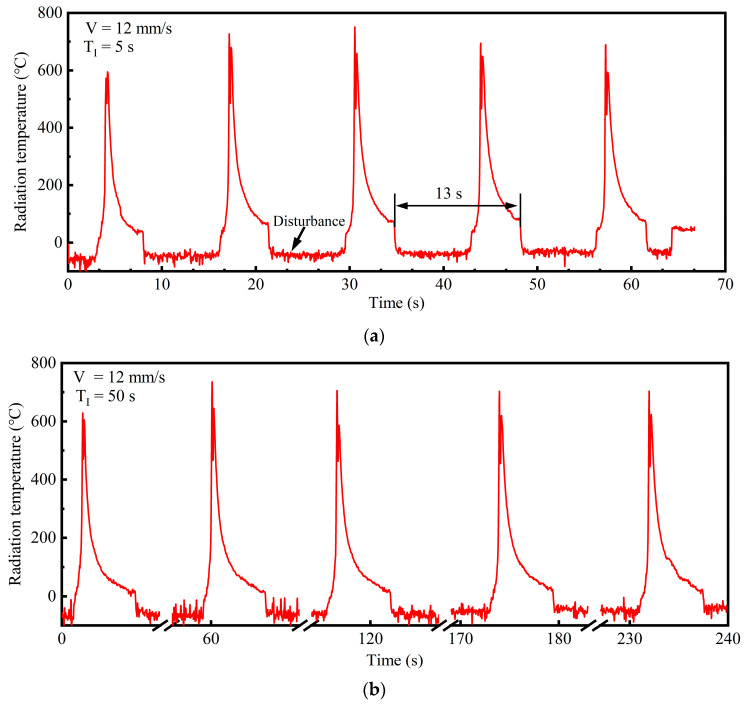
Thermal history and mean cooling rates of the melt pool surfaces. (**a**) Radiation temperature of Sample 2 layers, scanning speed (V) = 12 mm/s and interlayer dwell time (T_I_) = 5 s. (**b**) Radiation temperature of Sample 3 layers, scanning speed (V) = 12 mm/s and interlayer dwell time (T_I_) = 50 s.

Modification of process parameters to regulate the cooling rate represents an effective methodology for controlling the microstructure and resultant properties of the cladding layer [40]. With the same interlayer dwell time (T_I_) (5 s), the scanning speed (V) was increased to 12 mm/s for Sample 2 relative to Sample 1. Although the interlayer cooling interval was reduced from 17 s to 13 s, the mean cooling rate significantly increased, as shown in Table 5 and Figure 13a. The cooling rate reached 151 °C/s; the fourth layer increased by 45% compared to Sample 1. This indicates that the laser scanning speed predominantly affects the cooling rate. As the scanning speed increases, the laser line energy density (P/V) decreases and the melt pool interaction time drops, leading to faster solidification and a significant increase in the cooling rate. The reduced interlayer cooling interval provides a more insulating substrate for the subsequent cladding layer, which not only stabilizes the cooling rate but also substantially mitigates stress-induced cracks.

For Sample 3, the interlayer dwell time was extended to 50 s while maintaining the same scanning speed as Sample 2. Figure 13b shows that the thermal history profiles demonstrate minimal variations across all the layers. The mean cooling rate exhibits a slight decrease because heat accumulation descends according to the thermal gradients. Notably, the last layer almost shows no change in the cooling rate (Table 6). The findings indicate that extending the interlayer dwell time mainly weakens the insulation effect of the cladding process while marginally enhancing the melt pool surface cooling rate. In addition, the cooling rate at ambient temperature mainly depends on the substrate bulk and thermal conduction; thermal radiation and convection between the cladding layer and the ambient environment exhibit a limited impact on cooling rates. To accelerate the cooling process, active cooling strategies have been widely used in recent years, including substrate in situ cooling [28] and immersion cooling techniques [45].

**Figure 14:**
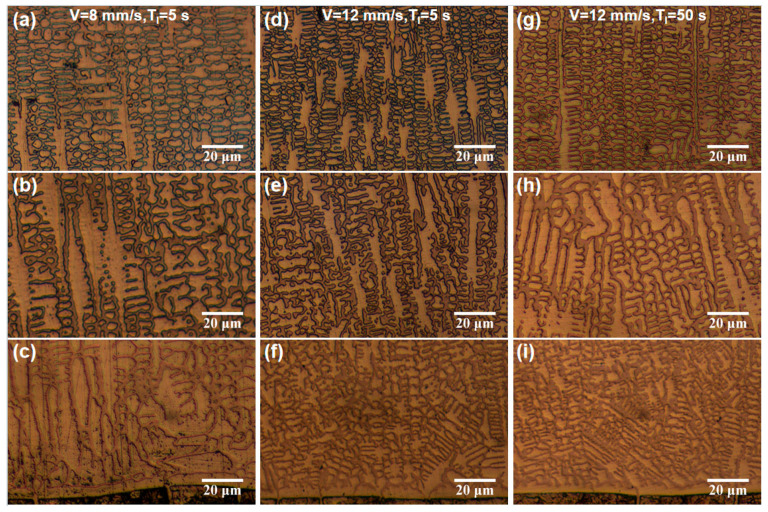
Comparative analysis of the effect of microstructure on cooling rate regulation. (**a**,**d**,**g**) Top regions of Samples 1~3. (**b**,**e**,**h**) Middle regions of Samples 1~3. (**c**,**f**,**i**) Bottom regions of Samples 1~3.

A comparative analysis of the microstructure characteristics among the samples is illustrated in Figure 14. At a lower scanning speed (7 mm/s) and shorter interlayer dwell time (5 s), Sample 1 exhibits prolonged laser-material interaction. The laser line energy density (P/V) increased the melt pool temperature, extending the grain growth duration and resulting in significantly coarser grains (Figure 14c). In Figure 14b, the middle region of Sample 1 experiences an insulation effect, reducing the temperature gradient. This thermal condition promotes grain refinement while avoiding excessive cooling. Conversely, the top region demonstrates higher cooling rates and undercooling degrees than the middle region, further enhancing grain refinement (Figure 14a). When the scanning speed increases from 7 mm/s to 12 mm/s, P/V reduces significantly and results in a temperature decrease and solidification acceleration of the melt pool. The resulting high cooling rate effectively suppresses grain growth. Therefore, Samples 2 and 3 exhibit significant grain refinement across all regions relative to Sample 1, with the most pronounced effect observed at the bottom of the cladding layer (see Figure 14c,f,i). Extending the interlayer time from 5 s to 50 s, the second to fifth layers of Sample 3 exhibit longer cooling times. This inhibits the heat accumulation and promotes the cooling rate. The bottom region of Sample 2 shows the same microstructure characteristics as Sample 3, while the middle region has finer grains corresponding to the higher cooling rate (Figure 14e,h). However, the top region of Sample 3 displays a finer microstructure than Sample 2, indicating that prolonging the interlayer dwell time can effectively enhance equiaxed dendritic crystal formation and refinement.

**Figure 15:**
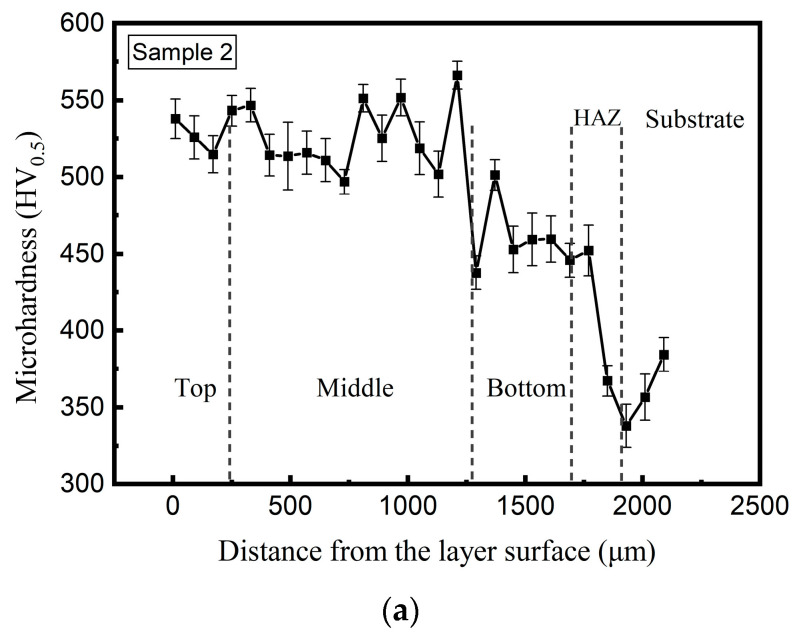

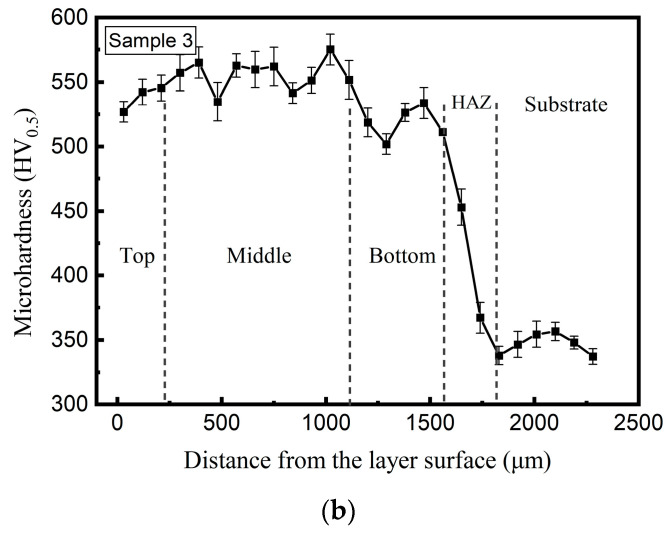
Comparative analysis of the effect of microhardness on interlayer dwell time (average ± σ). (**a**) Microhardness of Sample 2. (**b**) Microhardness of Sample 3.

As shown in Figure 15, the microhardness values of Samples 2 and 3 exceed 500 HV_0.5_ throughout the cladding layers, indicating significant hardness enhancement compared to Sample 1 (Figure 8). Notably, the top region of Sample 3 shows a higher hardness than Sample 2. The results coincide well with the microstructure characteristics of the samples in Figure 14.

Based on the performed analysis, the cooling rate can be effectively increased by either elevating the scanning speed or extending the interlayer dwell time, which consequently leads to significant improvements in both hardness and wear resistance properties. It should be emphasized that enhanced cooling rates may increase the crack susceptibility of the cladding layer, potentially inducing defects. However, Fe-based alloy materials have low crack sensitivity; there were no defects found in the samples, indicating the reliability of cooling rate enhancement. Furthermore, there were still difficulties in terms of closed-loop control of the transient cooling rate, and the underlying relationship between the cooling rate and the microstructure and properties of the cladding layer still requires in-depth research.

## 4. Conclusions

In this work, single-track muti-layer deposition experiments were conducted to study the geometric and microstructure characteristics of Fe-based alloy cladding layers. The radiation temperature of the melt pool was recorded online via an infrared camera, and the transient cooling rate was calculated according to the thermal history. Comparative analyses were performed to confirm the effectiveness of cooling rate enhancement in improving layer hardness. The main findings are as follows.

(1) Interlayer fusion lines appear distinctly due to coarsened grains formed by remelting of the pre-built layer. The geometric accuracy can be significantly improved by virtue of a predicted axis lifting amount (Z_d_), which ensures a constant standoff distance during each layer’s deposition.
(2) The Fe-based cladding layer exhibits different grain morphologies and sizes throughout the cross section. Planar grains form at the interface between the substrate and the cladding layer, and coarse columnar grains can be observed perpendicular to the bonding interface. Both dendritic and equiaxial grains coexist in the middle and top regions of the cladding layer, with finer grains on the top.
(3) Increasing the laser scanning speed or extending the interlayer dwell time can effectively enhance the cooling rate, resulting in appreciable improvements in coating hardness and wear resistance. The former approach demonstrates greater efficiency and effectiveness in enhancing the cooling rate. For large-part fabrication or large-area cladding, the deposition path should be rationally planned to increase the interlayer dwell time without compromising production efficiency.

## Figures and Tables

**Table 1:** Chemical composition of Fe-based alloy powder (wt%).

C	Cr	Ni	Fe	P	Mo	B	S
0.173	17.78	2.68	Bal.	0.013	0.90	0.86	0.004

**Table 2:** Particle size distribution and morphology characteristics of Fe-based alloy powder.

Size Distribution (μm)				Flow Rate (s/50 g)	Degree of Sphericity
D10	D50	D90	D97		
67.30	109.1	177.8	219.8	17.69	0.859

**Table 3:** Chemical composition of AISI 1045 (wt%).

C	Cr	Ni	Fe	Mn	Si
0.42	0.2	0.255	Bal.	0.65	0.3

**Table 4:** Process parameters of the three samples.

Sample	P (W)	V (mm/s)	F (g/min)	Z_d_ (mm)	T_I_ (s)	Q_p_ (L/min)	Q_S_ (L/min)	D_S_ (mm)
1#	2000	7	22.5	0.45	5	10	12	18
2#	2000	12	22.5	0.45	5	10	12	18
3#	2000	12	22.5	0.4	50	10	12	18

**Table 5:** Mean cooling rate of each cladding layer.

Layer No.	1st	2nd	3rd	4th	5th
**Mean cooling rate (°C** **/s)**	139	131	116	100	113

**Table 6:** Mean cooling rates of Samples 2 and 3.

Layer No.	1st	2nd	3rd	4th	5th
Mean cooling rate of Sample 2 (°C/s)	151	155	148	145	129
Mean cooling rate of Sample 3 (°C/s)	130	129	117	129	128

## Data Availability

The original contributions presented in this study are included in the article. Further inquiries can be directed to the corresponding author.
